# Translational Fidelity Decline in the Aging Oocyte and Embryo Development

**DOI:** 10.3390/ijms27062614

**Published:** 2026-03-12

**Authors:** Charalampos Voros, Fotios Chatzinikolaou, Georgios Papadimas, Ioannis Papapanagiotou, Aristotelis-Marios Koulakmanidis, Diamantis Athanasiou, Kyriakos Bananis, Antonia Athanasiou, Aikaterini Athanasiou, Charalampos Tsimpoukelis, Athanasios Karpouzos, Maria Anastasia Daskalaki, Christina Trakateli, Nana Kojo Koranteng, Marianna Theodora, Nikolaos Thomakos, Panagiotis Antsaklis, Dimitrios Loutradis, Georgios Daskalakis

**Affiliations:** 1 Department of Obstetrics and Gynecology, ‘Alexandra’ General Hospital, National and Kapodistrian University of Athens, 80 Vasilissis Sofias Avenue, 11528 Athens, Greece; aristoteliskoulak@gmail.com (A.-M.K.); diamathan16@gmail.com (D.A.);; 2 Laboratory of Forensic Medicine and Toxicology, School of Medicine, Aristotle University of Thessaloniki, 54124 Athens, Greece; fotischatzin@auth.gr (F.C.); loutradi@otenet.gr (D.L.); 3 Athens Medical School, National and Kapodistrian University of Athens, 15772 Athens, Greece; dr.georgepapadimas@gmail.com (G.P.); gpapamd@hotmail.com (I.P.);; 4 King’s College Hospital NHS Foundation Trust, London SE5 9RS, UK; kyriakos.bananis@nhs.net; 5 IVF Athens Reproduction Center, 15123 Maroussi, Greece; antoathan16@gmail.com (A.A.);; 6 3rd Department of Internal Medicine, Aristotle University, 54124 Thessaloniki, Greece; ctrak@auth.gr; 7 Fertility Institute-Assisted Reproduction Unit, Paster 15, 11528 Athens, Greece

**Keywords:** oocyte aging, translational control, maternal mRNA, ribosome fidelity, embryo development, assisted reproduction

## Abstract

Female reproductive aging is associated with a progressive decline in oocyte competence and reduced success in assisted reproductive technologies. While chromosomal abnormalities, mitochondrial dysfunction, and DNA damage have been extensively studied, these mechanisms do not fully explain developmental arrest in chromosomally euploid embryos or the variability in embryo competence. Human oocytes enter a transcriptionally quiescent state during meiotic maturation and rely almost entirely on the regulated translation of stored maternal messenger RNAs to support fertilization and early embryonic development until zygotic genome activation. In this context, translational fidelity becomes a critical determinant of proteome integrity and cellular function. Age-related alterations affecting ribosomal RNA integrity, transfer RNA modification, aminoacylation accuracy, and translational regulatory networks may impair the precision, timing, and coordination of protein synthesis. These defects can disrupt essential processes such as spindle assembly, cytoskeletal organization, and early cleavage dynamics, ultimately compromising embryo viability despite chromosomal normality. In addition, the follicular microenvironment, including redox balance, metabolic support, and signaling pathways, plays a crucial upstream role in maintaining translational integrity. This review integrates mechanistic evidence from molecular, cellular, and developmental studies to propose that progressive decline in translational fidelity represents a fundamental and previously underrecognized driver of reproductive aging. Understanding translational control as a central regulator of oocyte competence may provide new insights into unexplained IVF failure and support the development of novel biomarkers and therapeutic strategies aimed at preserving reproductive potential.

## 1. Introduction

As women age, the quality of their oocytes gradually declines. This restricts their capacity for procreation and diminishes their likelihood of success with assisted reproductive technologies. Clinical signs of this reduction encompass diminished fertilisation rates, suboptimal embryo development, an increased chance of miscarriage, and reduced live birth rates, especially following the late thirties [1]. In recent decades, researchers have investigated various cellular issues associated with ageing oocytes. Errors in chromosome segregation, loss of meiotic cohesion, mitochondrial malfunction, and cumulative DNA damage have become essential explanatory frameworks, influencing the current comprehension of reproductive aging [2].

These models are robust; they insufficiently address several enduring findings in both experimental and clinical contexts. Despite chromosomal tests indicating euploidy, a significant proportion of embryos derived from oocytes of older moms experience failure during the initial cleavage stages [3]. In contrast, developmental pathways may differ significantly across embryos with similar chromosomal arrangements, indicating that developmental potential cannot be solely deduced from genetic integrity [4]. The observed inconsistencies suggest that the age-related deterioration of oocyte function arises from abnormalities occurring downstream of the genome or irrespective of changes in DNA sequence [5].

The mammalian oocyte is a highly specialised cell that depends greatly on post-transcriptional control. During oocyte development, many messenger RNAs are synthesised and stored, frequently in a translationally inactive condition. Following the disintegration of the germinal vesicle and the transition of the cell into metaphase II, transcriptional activity significantly diminishes [6]. This indicates that the oocyte mostly relies on its preexisting transcriptome. Prior to the activation of the embryonic genome, maternal RNA and protein reserves furnish nearly all the support for meiosis, fertilisation, and initial embryonic development. The precision, timing, and specificity of protein synthesis predominantly dictate cellular functionality in these scenarios [7].

Transcripts are not the only determinant affecting the passive mechanism of protein synthesis in the oocyte. A complex network of RNA-binding proteins, translational initiation and elongation factors, ribosomal components, and signalling pathways that respond to stress and developmental signals meticulously regulates it [8]. During particular stages of maturation and early development, unique subsets of maternal transcripts are selectively activated or repressed within the oocyte’s dynamic translational program. Due to the absence of compensatory transcriptional responses throughout this formative period, even little alterations in this precisely calibrated system could significantly impact its functionality [9].

Aging influences numerous molecular processes that regulate translation, including redox equilibrium, metabolic stability, RNA integrity, and ribosomal activity. Age-related reductions in translational fidelity are linked to decreased cellular resilience and proteome instability across diverse cellular environments [10]. However, research is still insufficient to determine the extent to which similar pathways function in the oocyte. Most research in reproductive biology has focused on mitochondrial function, transcript levels, or epigenetic regulation. The molecular integrity of translation as a determinant of oocyte ageing has not been thoroughly investigated [11].

This review analyzes translational fidelity as a crucial yet underappreciated element affecting early embryonic development and the age-related decline in oocyte quality. By integrating data on oocyte maturation, RNA regulation, ribosome biology, and cellular aging, we suggest that a core mechanism connecting ageing to developmental failure is the progressive decline in translational precision and regulation. Examining oocyte aging via the prism of translational fidelity, alongside established genetic and mitochondrial models, provides insight into the enigmatic IVF outcomes observed in older mothers.

## 2. Literature Search and Analysis Methods

Our review, presented as a narrative and hypothesis-driven synthesis, concentrated on the molecular mechanisms connecting translational control to oocyte aging and a narrative approach was deemed more suitable than a systematic review methodology due to the conceptual character of the subject and the lack of a unique, clearly defined experimental endpoint. The main goal was to amalgamate mechanistic information from several experimental systems into a cohesive biological framework, rather than delivering an exhaustive quantitative overview of all current studies. The research concentrated on conserved molecular pathways regulating translational control during oocyte maturation and early embryonic development, integrating existing human studies alongside mechanistic investigations predominantly conducted in murine models.

Bovine models, in conjunction with murine and human studies, provide substantial translational insights into oocyte biology due to their physiological and developmental similarities to humans. Bovine oocytes are monovulatory, comparable in size to human oocytes, and demonstrate similar timing in meiotic maturation and initial embryonic cleavage. Bovine folliculogenesis and oocyte developmental capability more precisely represent human reproductive physiology than rodent models. Thus, bovine investigations offer substantial further evidence for clarifying translational control, oocyte competency, and early embryonic development in a clinically relevant mammalian context.

PubMed served as the principal database for a focused literature review. Throughout the composition of the work, iterative searches were conducted and refined to incorporate interconnected new concepts. Search terms encompassed combinations of oocyte, maternal mRNA, translation, translational regulation, ribosome, RNA-binding proteins, oocyte-to-embryo transition, embryonic arrest, and ageing. To include research on upstream modulators of translational processes, additional terminology pertaining to oxidative stress, metabolic control, and follicular microenvironment were included. The reference lists of the selected publications were manually scrutinised to find pertinent studies not included in the keyword searches.

Priority was given to original experimental research that clarifies the mechanistic features of translational regulation throughout oocyte maturation, fertilization, and early embryogenesis. The primary focus of the study was mechanistic investigations in murine models, which provide experimentally accessible systems for analyzing translational regulation during oocyte maturation and early embryogenesis. When possible, studies that used human oocytes and embryos were included to support translational interpretation and clinical relevance. These studies focused on conserved molecular pathways in mammalian reproduction. Only studies examining transcript abundance, rather than translation function, were included if they contributed to a broader understanding of regulatory concepts. Review articles were utilised to contextualise established biological principles and facilitate conceptual integration in domains characterised by complex regulatory frameworks encompassing multiple experimental systems. Review articles helped bring together what we already knew and fit translational regulation into the larger picture of oocyte biology and early embryonic development. When possible, original experimental research was used to back up specific mechanistic interpretations.

The selection of papers was based on their relevance to the core concept of translational integrity, rather than on established inclusion or exclusion criteria. Research prioritised ribosomal function, RNA-binding protein activity, processes of translational initiation and elongation, and signalling pathways that regulate oocyte protein synthesis. Experimental evidence associating translational alterations with functional outcomes, such as meiotic progression, cytoplasmic maturation, or early embryonic development, were selected. The objective was conceptual integration instead of statistical synthesis; therefore, formal quality assessment and meta-analytic methods were not utilised.

The chosen literature was subjected to qualitative analysis, and the results were interpreted in the context of established knowledge regarding cellular stress responses, oocyte biology, and reproductive ageing. Considering the distinct biological limitations of the oocyte, mechanistic similarities from research on other cell types were utilised where needed for interpretation. The objective of this integrative approach was to create a unified model that can direct future experimental and translational research, while highlighting the neglected relationships between translational regulation and reproductive ageing.

## 3. Biological Dependence of the Oocyte on Maternal Translation

The rigid temporal distinction between transcription and translation poses a significant biological challenge in the mammalian oocyte. Throughout the prolonged growth period, oocytes actively transcribe and amass a diverse array of maternal messenger RNAs that encode proteins essential for meiotic maturation, fertilisation, and initial embryonic development. These transcripts are produced during the development of human oocytes and remain in the cytoplasm until meiosis and the onset of embryonic development. As oocytes resume meiosis and progress to metaphase II, transcriptional activity markedly declines and becomes functionally insignificant, rendering the oocyte highly dependent on maternally stored mRNAs and meticulously regulated translational mechanisms to support meiotic advancement and initial embryonic development. This transcriptional silencing demonstrates chromatin condensation and the comprehensive inhibition of RNA polymerase II activity. From this juncture onwards, the oocyte relies on the regulated translation of pre-existing RNA pools, whose integrity and functional availability were established before to meiotic resumption, to implement intricate and precisely coordinated developmental programs without de novo transcription [6,7,12,13,14]. Table 1 describes the most important studies on translational regulation during oocyte maturation, the transition from oocyte to embryo, and the transition from maternal to zygotic. This is done to systematically bring together the different experimental methods and developmental contexts that were discussed above.

The oocyte differs from most other cell types, as it does not transcribe RNA, hence relying significantly on translation control mechanisms. Somatic cells can modify their transcriptional output in reaction to stress or developmental signals, thereby compensating for issues related to protein synthesis or degradation [27]. In contrast, the oocyte relinquishes this ability to adapt following the reinitiation of meiosis. Thus, only post-transcriptional processes that regulate transcript selection, translational timing, and translational efficiency may control protein synthesis. Any interruption affecting these mechanisms influences the following developmental phases and cannot be remedied by compensatory transcription. As a result, translation fidelity and robustness shift from secondary regulatory characteristics to primary factors influencing meiotic competence and developmental potential [28].

Maternal mRNAs are not retained as dormant templates awaiting random translation. Rather, they constitute ribonucleoprotein complexes that meticulously regulate transcript stability, localisation, and translational competence [29]. Short poly(A) tails and associations with specific RNA-binding proteins that inhibit ribosome recruitment prevent the translation of many transcripts. These complexes enable the oocyte to retain transcripts for an extended period in a quiescent yet responsive condition [30]. Meiotic development initiates cytoplasmic polyadenylation activities that are meticulously planned, activating certain subsets of maternal transcripts by elongating poly(A) tails and facilitating translational initiation. This technique allows for fast and coordinated changes in protein synthesis without changing transcript abundance, which makes it possible to obtain temporal accuracy that would be challenging to get through transcriptional regulation alone [31].

Oocyte maturation involves a very dynamic temporal pattern of translation marked by distinct phases. Notable developmental events, including as germinal vesicle breakdown, spindle formation, chromosomal alignment, and advancement to metaphase II, are linked to distinct phases of translational activation and repression [32]. These translational waves influence clusters of functionally related genes, including those that regulate vesicular movement, alter actin morphology, modify microtubule dynamics, and govern the cell cycle. They do not encompass the entire globe. Maternal mRNAs are utilised for protein synthesis exclusively in specific contexts and temporalities. Even if it impacts a limited number of transcripts, disrupting these closely associated translational programs could destabilise the broader regulatory network and diminish oocyte competence [29].

During fertilisation, reliance on maternal translation intensifies. The early embryo experiences swift cleavage divisions after sperm entrance, without much transcriptional activity. Maternal proteins and residual maternal transcripts are the primary determinants affecting developmental advancement until the maternal-to-zygotic transition, signifying the activation of the embryonic genome [33]. Protein reservoirs formed during oocyte maturation are crucial for centrosome operation, cytoskeletal rearrangement, cell cycle progression, and initial patterning. Thus, any qualitative or quantitative inadequacy in these proteins is directly conveyed to the embryo and cannot be remedied until zygotic transcription commences [34].

In the absence of transcriptional buffering, the consequences of translational disturbances are exacerbated. Errors in protein synthesis may occur if essential regulators are improperly translated, if translation occurs at inappropriate times relative to cell cycle events, or if elongation is less precise [35]. These abnormalities are particularly significant as numerous cellular activities in the oocyte and early embryo rely on the correct quantity and configuration of proteins. Alterations in protein quantity or functionality can significantly impact actin-mediated cytoplasmic reorganisation, kinetochore–microtubule attachment, and spindle assembly. Minor alterations in the optimal protein composition can disrupt these processes, resulting in anomalous cleavage patterns, delayed growth, or even a total cessation of development [36].

The translational apparatus must exhibit significant long-term stability because to its reliance on maternal translation. It is essential for ribosomes, transfer RNAs, and other translation factors to function well over extended periods while withstanding cumulative metabolic and oxidative stress. In contrast to growing somatic cells, oocytes are unable to rapidly replace damaged components through transcription or dilute them via division. As a result, molecular damage to translation factors, tRNA integrity, or ribosomal RNA may insidiously accumulate over time. This type of injury may not entirely inhibit translation, but it might gradually diminish its accuracy or efficiency, adversely affecting developmental competence in a manner contingent upon age.

## 4. Molecular Architecture of Translational Fidelity in Oocytes

Translational fidelity refers to the capacity of the translational machinery to synthesise proteins from messenger RNA templates with precision, efficiency, and in the appropriate temporal sequence. The lack of transcriptional compensation and the extended interval between mRNA production and protein synthesis provide distinct biological importance to this process in the oocyte [37]. In this case, translational fidelity is regulated by the integrated activity of ribosomes, transfer RNAs, translation initiation and elongation factors, and regulatory signalling pathways, rather than by a single molecular entity [38].

The ribosome, a ribonucleoprotein complex central to the translational apparatus, is crucial for the precise decoding of mRNA due to its structural and functional integrity. Ribosomal RNA (rRNA) constitutes the catalytic framework of the ribosome. This directly influences peptidyl transfer, decoding, and ribosomal dynamics during elongation. Ribosomes in oocytes must function for extended durations often years in humans since they cannot be replenished through cell division [39]. Due to the longevity of rRNA, it is particularly susceptible to cumulative molecular degradation. Oxidative modifications, base lesions, and structural alterations in rRNA can gradually impair ribosomal activity without entirely halting translation. This may alter the precision of decoding or the rate of elongation. These errors may result in the synthesis of proteins with modified folding characteristics or amino acid compositions by impairing translational accuracy rather than translational efficiency [40].

Transfer RNAs (tRNAs) significantly influence translational fidelity. Accurate protein synthesis necessitates the proper aminoacylation of tRNAs and the right pairing of codons with anticodons during elongation. tRNAs experience significant post-transcriptional alterations that are crucial for preserving translational efficiency, ribosome stability, and decoding precision [41]. Maintaining the stability of tRNA modification patterns in the oocyte over extended durations and across developmental transitions is crucial. Age-associated alterations in tRNA modification enzymes or substrate accessibility may disturb these patterns, heightening the probability of codon misreading or ribosomal stalling. In the oocyte, where the stoichiometry and structural integrity of vital proteins are meticulously maintained, even infrequent errors in amino acid incorporation can have excessive effects [42].

Translation initiation serves as a crucial regulatory checkpoint that integrates developmental cues with translation output. To guarantee that only particular subsets of maternal transcripts are translated at specific phases, initiation in oocytes is rigorously controlled [43]. This selectivity results from specialized initiation factors and interactions between RNA-binding proteins and the 5′ untranslated regions of transcripts. Alterations in the availability or activity of initiation factors can disrupt the equilibrium between translationally active and repressed transcripts. This may result in incorrect timing or amounts of protein production [44]. Significantly, initiation errors can disrupt developmental systems reliant on precise temporal coordination by modifying the relative abundance of individual proteins rather than diminishing overall protein synthesis [35].

In the oocyte, the integrity of elongation is also crucial, especially during phases of heightened translational demand, such as meiotic development and early embryonic cleavage. The velocity and precision of elongation are contingent upon the functionality of elongation factors, the accessibility of tRNA, and the structural integrity of the ribosome [45]. Disruptions impacting elongation kinetics might result in ribosomes ceasing function, completing prematurely, or increasing error rates. Such flaws frequently activate quality control mechanisms that degrade aberrant nascent polypeptides in somatic systems. However, the oocyte may lack the capacity to rectify erroneous proteins, leading to their accumulation and obstruction of critical cellular functions [46].

Signalling circuits that regulate translation further complicate the molecular structure of translational fidelity. The PI3K-AKT-mTOR pathways integrate food availability, energy status, and developmental signals with translational output. In oocytes, these pathways must be meticulously adjusted to ensure a balance between metabolic capability and protein synthesis [47]. Dysregulation of translational signalling can intensify oxidative stress and further undermine translational fidelity by disturbing the connection between protein production and cellular homeostasis. Notably, signaling-mediated alterations in translation frequently impact certain subsets of transcripts, underscoring the idea that translational dysregulation in oocytes is selective rather than widespread. Thus, the translational fidelity framework in oocytes is intrinsically delicate [48]. Stable ribosome components must be maintained over time, with precise regulation of initiation and elongation, accurate modification and charging of tRNA, and the capacity to integrate signalling inputs in a metabolically constrained environment. Oocytes cannot rely on cell division or transcriptional renewal when translational integrity is impaired, unlike somatic cells. Over time, even minor age-related issues affecting any component of this system may gradually diminish translational fidelity [49].

## 5. RNA-Binding Proteins as Gatekeepers of Selective Translation

RNA-binding proteins (RBPs) control the stability, location, and ability of maternal transcripts to be translated. This, in turn, controls selective translation in the oocyte. These proteins serve as molecular gatekeepers by determining which mRNAs remain inactive, which become translationally active, and the precise timing of translation [50]. RBPs have a job that goes beyond controlling gene expression. They are also important for the identity and function of cells in the oocyte, where transcriptional activity is low after meiotic resumption. Their activity not only counts the number of transcripts, but it also makes sure that protein synthesis follows a certain developmental logic [51].

When compared to somatic cells, this reliance on RBPs shows a big change in regulatory strategy. Transcriptional programs in proliferating cells can be swiftly modified to respond to changes in environmental stress or protein synthesis [52]. On the other hand, the oocyte needs to rely on post-transcriptional mechanisms to get the same level of regulatory flexibility. RBPs give this flexibility by controlling translational repression, activation, and buffering during developmental changes. They make a complicated set of rules that lets them change the proteome in very specific ways without having to make new transcription. This is possible because of combinatorial binding and interactions that depend on the situation. So, when this network is broken, it can have effects that go beyond just some transcripts and could even hurt the whole translation program [53].

Cytoplasmic polyadenylation element-binding protein 1 (CPEB1) is a key part of this regulatory system. CPEB1 directly links translational repression to activation by attaching to certain sequence elements in the 3′ untranslated regions of target mRNAs. You can do this by changing the length of the poly(A) tail [54]. Transcripts associated with CPEB1 are generally not found in active polysomes and have short poly(A) tails in immature oocytes. In this repressed state, transcripts are protected, and the production of proteins begins. After meiosis starts again, CPEB1 goes through changes that happen after translation, such as phosphorylation by kinases that are controlled by the cell cycle. These changes change how it works with complexes that add and remove polyadenylation [27].

These changes start cytoplasmic polyadenylation, which makes the poly(A) tail longer and brings in more factors that start translation. This process is very picky, it only affects certain groups of transcripts, which is very important [55]. It does not cause global translational activation. Proteins produced via CPEB1-dependent activation are frequently essential for the regulation of the cell cycle, chromosome segregation, and spindle formation processes that require exact temporal coordination. So, even if the amount of transcripts stays the same, changes in how CPEB1 works can make proteins at the wrong time [12]. Minor alterations in the quantity, phosphorylation dynamics, or RNA-binding affinity of CPEB1 in ageing oocytes may lead to asynchronous translation and diminished meiotic fidelity.

The regulatory logic that controls CPEB1 function illustrates a broader principle for translational control in oocytes: repression and activation are not opposing extremes but rather interrelated states that occur sequentially [56]. Translational repression not only stops transcripts from being made, but it also keeps them responsive and safe from damage. Activation must occur within a specific timeframe to ensure that protein synthesis meets developmental requirements. Any kind of interruption can be bad. If you wait too long to activate, there might not be enough protein at key points. If you activate too soon, you might use up protein stores or mess up the stoichiometric balance. This makes the repression–activation cycle more likely to be affected by changes that happen with age [57].

DAZL (Deleted in Azoospermia-Like) is the second most important group of RBPs. It has a different but complementary role in oocyte translation. DAZL can either stop or start translation by binding to certain motifs in target mRNAs [13]. This depends on the developmental context and the partners it interacts with. DAZL helps keep transcripts stable and stops them from being made when oocytes are still growing. This keeps the mother’s mRNAs safe until they are needed. DAZL helps ribosomes attach to transcripts that are important for meiotic progression and developmental competence as maturation happens by being involved in translational activation [19].

DAZL changes how ribosomes are loaded by interacting with parts of the machinery that starts translation, not how poly(A) tails move. This method allows DAZL to change how quickly translation happens without changing how stable the transcript is. DAZL also works with other RBPs, such as CPEB1 [58]. This shows that coordinated RBP assemblies, not just one factor, control translation in the oocyte. Changes in DAZL expression, localisation, or RNA-binding specificity can have a big effect on the translational status of many transcripts that are linked to similar biological processes. Even minor reductions in DAZL-mediated translational activation may significantly impact proteins essential for cytoskeletal organization and checkpoint regulation in ageing oocytes [59].

Y-box binding protein 1 (YBX1) adds another level of translational control by stabilising and buffering maternal transcripts. YBX1 makes messenger ribonucleoprotein complexes that keep transcripts from breaking down by binding to mRNAs in many different ways. This buffering ability is very important in the oocyte because the transcripts need to stay intact and functional for a long time without being renewed. YBX1 preserves the developmental potential of the oocyte during extended periods of arrest by maintaining transcripts in an inactive state, primed for translation [60].

YBX1 is responsible for stabilising transcripts and controlling translation, as well as for cellular signalling and stress responses. YBX1’s function is influenced by post-translational modifications and interactions with signalling pathways that indicate metabolic and redox status. YBX1 can change how easy it is for the translational machinery to get to transcripts when the cell’s environment changes [61]. As YBX1 changes as we get older, it may make it harder for the oocyte to act as a buffer. This could make it more likely for transcripts to break down or be translated incorrectly. These changes might not be clear when looking at transcript abundance, but they can still change the proteome’s makeup and the timing of development [62].

Translation is selective because of a complex system of rules that includes the coordinated actions of CPEB1, DAZL, YBX1, and other related RBPs. RBP networks that react to developmental signals include polyadenylation dynamics, ribosome recruitment, transcript stabilisation, and translational repression. This structure makes sure that proteins are made in the right order and at the right levels and lets the oocyte make functional diversity from a static transcriptome. This selectivity is important because it lets the oocyte meet complicated developmental needs without needing transcriptional input [19].

From a molecular perspective, this RBP-centric regulatory network represents a significant vulnerability during reproductive ageing. For RBPs to work, they need to be in the right place in the cell, have the right changes made to them after translation, and stay structurally sound. Oxidative stress that comes with age, changes in kinase signalling, and changes in protein turnover may make RBP work less well without lowering transcript levels [63]. These issues could create a disparity between protein supply and developmental requirements by dissociating transcript availability from translation output. These mismatches may make oocyte competence slowly worse over time.

## 6. Age-Related Perturbations of Translational Control

Reproductive ageing impacts the egg through the gradual accumulation of molecular stress rather than through abrupt, catastrophic occurrences. Translational control depends on macromolecular assemblies that must operate for prolonged periods, making it especially vulnerable to this process. Translation consistently occurs during meiotic maturation and the initial phases of development. This differs from transcriptional regulation, which may be unnecessary until gene expression is required. Consequently, even minor age-related impairments in translational machinery can immediately affect its functionality. In the oocyte, such issues cannot be rectified or mitigated due to the absence of transcriptional compensation upon meiotic restart. Instead, they proceed directly to the proteome. Table 2 depicts the main age-related changes that affect translational fidelity and proteome integrity. These changes show how reproductive aging affects translational control at different molecular levels.

The fundamental components of translational control exhibit considerable sensitivity to ageing, highlighting their persistent traits. In human oocytes, ribosomes, RNA-binding proteins, translation factors, and transfer RNAs must maintain structural integrity and functional coordination across decades of dormancy. Throughout this period, the molecular equilibrium within the body gradually alters due to the accumulation of metabolic waste products, reactive oxygen species, and fluctuations in nutrition availability [64]. Oocytes are unable to repair damaged components through replication or fragmentation, unlike proliferating cells. Consequently, transient or insignificant translational mistakes in other cell types may accumulate in the oocyte unnoticed. They attain biological significance solely when the requirement for translation escalates during meiosis [65].

Oxidative stress is a prevalent consequence of ageing on translational regulation. The ageing ovarian follicle accumulates increased quantities of reactive oxygen and nitrogen species due to changes in follicular metabolism, redox buffering, and mitochondrial inefficiency [66]. Due to its prevalence, structural complexity, and infrequent alterations, ribosomal RNA is particularly susceptible to oxidative modification. Oxidative damage to rRNA can slightly alter the conformation of ribosomes, hence affecting the efficiency of peptidyl transfer and the morphology of the decoding site. These alterations may increase the frequency of frameshifting, ribosomal stalling, or decoding errors. This diminishes translation accuracy; however, it does not invariably prevent it from occurring [67].

Oxidative damage to ribosomes is particularly significant in oocytes because of the difficulty in rectifying errors that occur during translation. Quality control mechanisms are less operational in the egg and early embryo, resulting in the potential oversight of misfolded proteins or incorrectly incorporated amino acids [68]. Aberrant proteins may persist within cells and inhibit critical physiological activities. Proteins that facilitate spindle formation, associate with kinetochores, and organise the cytoskeleton exhibit heightened sensitivity to structural alterations. Their function is contingent upon the efficacy of their folding and their interactions with one another. Minor errors in translation that impact these proteins could jeopardise the fidelity of meiosis and disrupt the higher-order structure of cells [69].

Aging may induce oxidative stress, which can alter RNA-binding proteins that regulate selective translation. Proteins such as CPEB1, DAZL, and YBX1 require well specified tertiary structures and post-translational modifications to maintain their RNA-binding specificity and regulatory roles [70]. Oxidative modifications to amino acid residues or the disruption of phosphorylation-dependent interactions can alter their affinity for target transcripts or their efficacy in recruiting translational machinery. These modifications may not entirely inhibit RBP’s functionality, but they could complicate temporal regulation. This may result in translational activation occurring prematurely, belatedly, or not occurring at all. Such minor issues may suffice to impede development in a system reliant on exact time [71].

Another significant manner in which ageing influences translational control is by disrupting the body’s energy use. Oocytes from older individuals typically produce reduced ATP, utilise alternative substrates, and possess mitochondria with diminished functionality. Translation requires substantial energy during ribosomal recycling and elongation processes [72]. Adequate ATP levels are essential for resolving translational pauses, insufficient ATP may lead to increased ribosomal stalling and diminished elongation rates. These effects may exacerbate decoding errors and jeopardize protein synthesis, particularly during periods of high translation demand [73].

The metabolic status of the cell significantly influences the efficiency of tRNA charging and the availability of amino acids. Ageing may diminish the accuracy of aminoacylation by altering amino acid pools, affecting the activity of redox-sensitive aminoacyl-tRNA synthetase, or modifying tRNA enzymes [74]. Ribosomes may halt prematurely if tRNAs are inadequately charged or incorrectly acylated. This increases the likelihood of errors occurring during translation. Such errors may significantly affect proteins that must uphold exact stoichiometric balance or experience swift alterations during meiotic transitions in the oocyte, when protein synthesis necessitates high precision [75].

Nutrient-sensing signalling mechanisms that influence translational regulation are altered with ageing. The PI3K-AKT-mTOR pathway integrates data on energy status, amino acid supply, and growth cues to regulate the initiation of translation and ribosomal function [76]. In oocytes, this system must be precisely regulated to equilibrate metabolic capacity with protein production. Age-related dysregulation of mTOR signalling may result in inadequate translational activation, possibly decoupling protein synthesis from available resources. Insufficient activation will impede the cell’s ability to produce adequate proteins essential for spindle formation and the progression of the cell cycle. Conversely, over activation can exacerbate oxidative stress and deplete the body’s finite energy reserves [77].

The mechanisms of translational suppression are significantly influenced by ageing. Transcripts are securely preserved, and premature protein synthesis is inhibited by the actively regulated condition referred to as translational repression [78]. To sustain repression, consistent patterns of post-translational modification, efficient RNA-binding proteins, and intact ribonucleoprotein complexes are essential. As individuals age, these systems may become less stable, resulting in diminished ectopic translation and reduced repression efficacy. If repression and activation cycles become uncoupled, premature synthesis can disrupt proteome equilibrium and hinder the proper sequencing of developmental program activation [79].

These alterations occur increasingly with time due to the prolonged lifespan of the oocyte. For decades, human oocytes have remained in prophase I, exposing translational machinery components to continuous oxidative and metabolic stress. Accumulated damage may not become apparent as malfunction until there is an increased translational demand during meiotic restart. At this juncture, the oocyte must rapidly synthesise several proteins that function together. Consequently, pre-existing translation mistakes may become rate-limiting factors, hindering early development or entirely obstructing maturation. It is essential to recognize that age-related translational disruptions are improbable to result in uniform reductions in protein synthesis. Instead, they produce proteins that require precise stoichiometry, timing, or localisation, rendering them vulnerable in a manner that only impacts them. Certain mechanisms predisposed to these issues include spindle assembly, chromosomal segregation, actin-mediated cytoplasmic remodelling, and checkpoint signaling.

## 7. Translational Dysregulation and Early Embryo Development

The quality of early embryonic development is directly correlated with the integrity of the maternal proteome established during oocyte maturation. The embryo functions within a closed molecular system prior to the activation of the embryonic genome [80]. Proteins made by the mother and leftover maternal transcripts power almost all biological functions. This developmental constraint directly and inescapably associates the developmental competence of the resultant embryo with the translational processes occurring during oocyte maturation [81]. As a result, translational fidelity defects that occur in the oocyte are incorporated into the molecular framework that controls cleavage divisions, cytoskeletal organization, and the first lineage determinations, instead of being temporary problems that only happen at one stage of the cell cycle [82].

This continuity is especially important because early embryos don’t have the same backup systems that differentiated cells do. Proteins essential for DNA replication, mitotic spindle assembly, centrosome function, and cytokinesis must be present in adequate quantities and functional conformations from the outset. Changing pathways, increasing transcription, or changing proteins can be hard to make up for. Because of this, translational errors that happen during oocyte maturation can last through many stages of development. These flaws can have effects that get worse over time and may not show up until later stages, like when the blastocyst forms or when the cells get compacted.

During the first cleavage divisions, embryonic cells go through a fast and synchronised cell cycle. This is clear because the gap phases are shorter, the checkpoints are less strict, and the entry into mitosis is faster [83]. These divisions put a lot of pressure on the availability and function of proteins that come from the mother and control DNA replication licensing, spindle assembly, chromosome segregation, and cytokinesis. Transcription is either very low or not happening at all during this time, so the embryo can’t change the levels of proteins when it is stressed or out of balance [84]. Consequently, even minor quantitative or qualitative deficiencies in critical regulators can result in significant developmental issues such as delayed cleavage, abnormal blastomere symmetry, multinucleation, or total developmental arrest. These results frequently indicate threshold effects rather than a complete loss of molecular function. The effective concentration of important proteins may fall below the level needed for reliable cell cycle transitions if translation isn’t working right, elongation fidelity isn’t high enough, or initiation timing changes [85]. Minor alterations in protein quantity or activity can significantly influence cleavage kinetics, cell cycle coordination, and embryo viability in early embryos, where timing and synchrony are intricately connected to developmental success.

Translational dysregulation impacts spindle organization in the initial mitotic divisions, a critical and sensitive phase. The synthesis and stoichiometric balance of tubulin isoforms, microtubule-associated proteins, motor proteins, centrosomal scaffolds, and regulatory kinases must all work together for the spindle to form correctly [86]. These parts need to be made in the right amounts and sent to certain parts of the cell at certain times. Errors in translation that mess up spindle architecture can lead to chromosomes being in the wrong place, chromosomes that are lagging, merotelic attachments, or problems with anaphase progression [87].

It is important to note that chromosomal abnormalities at fertilisation may not be necessary for the development of spindle-related defects. An embryo may be chromosomally euploid, yet its protein supply may be insufficient or malfunctioning, thereby impeding the proper completion of mitosis. This discrepancy indicates a significant issue with assessments of embryonic quality predicated on the genome [88]. Translational dysregulation elucidates the mechanisms underlying mitotic failure in embryos that, despite appearing genetically normal, demonstrate suboptimal developmental trajectories.

Translational defects considerably impact extensive cytoskeletal remodelling processes that influence early embryo architecture, transcending spindle assembly. Actin-mediated dynamics regulate the alignment of the cleavage plane, the arrangement of the blastomeres, the densification of the cells, and the determination of cell polarity [89]. To make these things happen, actin regulators, scaffolding proteins, and small GTPase signalling components must be made, moved to the right places, and broken down in a very controlled way. Cytoskeletal plasticity can become less stable if there is translational insufficiency or mistiming that affects these factors. This can lead to uneven cleavage patterns, changes in how cells stick together, and problems with compaction. It is necessary to note that problems with the cytoskeleton often come before clear signs of developmental failure, such as changes in shape [90]. Early embryos may harbour concealed protein-level anomalies that gradually disrupt their structural organization, despite initially appearing normal externally. In embryos at the cleavage stages, early translational disruptions can lead to delayed but significant effects on developmental outcomes, as the integrity of the cytoskeleton is crucial for subsequent lineage allocation and blastocyst morphogenesis [91].

The maternal-to-zygotic transition (MZT) is a crucial developmental stage during which control shifts from maternal products to zygotic gene expression. To make this change go smoothly, the embryonic genome needs to be turned on at the right time, and maternal transcripts and proteins need to be removed in a coordinated way [92]. Translational dysregulation can disturb this balance via multiple mechanistic pathways. Transcript clearance might be delayed because of insufficient protein synthesis linked to maternal mRNA decay pathways, thus extending maternal influence beyond the specified developmental timeframe. On the other hand, ineffective zygotic genome activation may happen when there aren’t enough transcriptional activators, chromatin remodelers, or basal transcription machinery [18]. The unusual persistence of maternal proteins generated during oocyte maturation may impede the formation of zygotic regulatory networks. Proteins that help with early cleavage may become inhibitory as soon as zygotic transcription starts. This means they need to be broken down or replaced quickly. Thus, translational defects that modify the timing, quantity, or stability of maternal proteins can hinder developmental reprogramming, resulting in an incomplete or unsuccessful MZT and subsequent developmental arrest [93].

Furthermore, translational dysregulation significantly influences early embryonic epigenetic regulation. In the early stages of development, the mother makes most of the enzymes needed for DNA methylation, histone modification, and chromatin remodelling [94]. The number and activity of oocytes are influenced by the extent of translation occurring during their maturation. Mistakes in making or controlling these enzymes can change how easy it is for chromatin to be accessed, how well it can be transcribed, and how it can be used to determine lineage. These epigenetic disruptions could affect implantation potential and long-term developmental stability, even after the early cleavage stages [95].

From a kinetic standpoint, translational defects often present as developmental delays rather than abrupt cessation. Embryos derived from oocytes exhibiting suboptimal translational fidelity may experience delayed or irregular cleavage timing, potentially indicating that proteins are either unavailable or functioning inadequately [96]. These kinetic anomalies have been shown to predict insufficient developmental competence and reduced implantation potential and appear before morphological anomalies, indicating that translational defects have an early and cumulative effect [97]. Translational dysregulation has a greater effect on early embryos due to their limited proteostatic capacity. In the early stages of development, quality control pathways that break down proteins that are not working right or are misfolded are not very well developed. This is why abnormal proteins made during oocyte maturation may stay around for a few cleavage divisions [98]. Their combined effects can slow down development, change the structure of cells, and mess with signalling pathways long before the embryo reaches the blastocyst stage. Even in the absence of detectable aneuploidy, these mechanisms collectively offer a coherent molecular explanation for the frequent occurrence of embryo arrest in IVF cycles linked to advanced maternal age. Translational dysregulation reconciles genetic normalcy and functional failure, elucidating why developmental competence is not consistently restored by chromosomal selection-only strategies such as PGT-A. Such methods cannot completely correct abnormalities occurring during oocyte maturation if they do not address defects inherent in the maternal proteome.

Porcine embryos represent a crucial translational model for human embryonic development, in conjunction with the models found in mice and cattle. In contrast to mouse models, which demonstrate earlier transitions, swine embryonic genome activation occurs during the 4- to 8-cell stage, closely mirroring human embryos. Furthermore, pig embryogenesis more closely resembles human development in terms of the temporal dynamics of blastocyst formation, lineage segregation, and the establishment of pluripotency. Porcine models are especially beneficial for examining translational control, proteome integrity, and developmental competence within a physiologically appropriate mammalian framework because of these similarities. Thus, mechanistic insights gained from porcine embryos enhance the translational relevance of molecular models of human embryonic development and supplement those obtained from mouse research.

## 8. Influence of the Follicular Microenvironment on Translational Integrity

The oocyte matures within the specialised and dynamic milieu of the ovarian follicle, rather than in isolation. Throughout folliculogenesis, the granulosa and cumulus cells encasing the oocyte supply metabolic substrates, signalling factors, and redox buffering [99]. This microenvironment significantly influences oocyte competence by regulating cellular stress responses, energy availability, and molecular equilibrium. The follicular environment can significantly disrupt translational integrity due to its reliance on the long-term stability of ribosomes, RNA-binding proteins, and signalling pathways [100].

The redox status of the follicular microenvironment is of paramount importance. The follicle continuously produces reactive oxygen and nitrogen species because of steroidogenesis, inflammatory signalling, and mitochondrial activity [101]. In youthful, healthy follicles, antioxidant mechanisms in both somatic cells and the oocyte meticulously regulate these reactive species. Redox homeostasis progressively declines with advancing maternal age or in pathological states [102]. Elevated oxidative stress in follicular fluid can directly impact macromolecules in oocytes, including ribosomal RNA, transfer RNAs, and translational regulators. This type of damage can diminish translation accuracy and coordination, although it may not enough to lethally impact the cell or alter its morphology in a readily observable manner.

Oxidative stress within the follicular milieu can indirectly influence translational regulation by altering the communication between oocytes and somatic cells. Granulosa and cumulus cells metabolise glucose and amino acids to produce the essential components required by oocytes for protein synthesis and energy production [103]. Oxidative damage to somatic cells might impair metabolic activities by decreasing the supply of pyruvate, amino acids, and reducing equivalents to the oocyte. These limitations may restrict translational capacity and heighten susceptibility to elongation mistakes or ribosomal stalling during periods of elevated translational demand, as translation is an energy-intensive activity intricately connected to metabolic status [104].

The availability of nutrients in the follicular milieu and the equilibrium of redox significantly influence the efficacy of translation. The amino acid composition of follicular fluid reflects the activity of local granulosa cells and the general metabolic state. The availability of amino acids may be influenced by ageing, metabolic disorders, or alterations in follicular vascularization [105]. This can alter the efficacy of aminoacyl-tRNA synthetase and the charging of tRNA. When amino acid pools are deficient or imbalanced, the likelihood of erroneous translation and incorrect codon reading increases. Such disturbances can significantly impact proteins that require stringent stoichiometric balance or rapid synthesis during meiotic transitions in the oocyte, where protein synthesis must be highly accurate [106].

The follicular microenvironment modifies the processes of translational control through juxtacrine and paracrine signalling pathways. Granulosa and cumulus cells secrete hormones, growth factors, and cytokines that initiate signaling cascades within the oocyte. These cascades ultimately converge onto the translational apparatus [107]. The PI3K-AKT-mTOR pathway integrates signals related to nutrition, energy, and growth to regulate the initiation of translation and ribosomal activity. Disruption of these pathways in the follicle, whether due to ageing or alterations in endocrine signaling, can decouple translational output from cellular capacity [108]. Inadequate signalling may impede the synthesis of proteins essential for meiotic completion, whereas excessive activation may exceed metabolic thresholds for protein synthesis, exacerbating oxidative stress. Moreover, it significantly influences both the magnitude and specificity of translation. RNA-binding proteins that respond to extracellular cues by modulating protein synthesis and transmitting signals regulate translation specific to particular transcripts. Altering follicular signaling may modify the equilibrium between translational repression and activation. This may render it an inappropriate moment to synthesise proteins crucial for development. This type of timing error can disrupt the sequence of events required for synchronized meiotic development and cytoplasmic maturation, although it may not alter the total protein quantity [109].

Inflammatory signalling within the follicular environment serves as an additional regulatory mechanism affecting translational integrity. There is a growing consensus that low-grade inflammation is a hallmark of ovarian ageing [110]. Follicular fluid comprises inflammatory mediators and cytokines that can initiate stress-responsive signalling pathways in both somatic and oocyte cells. These routes may alter ribosomal components, RNA-binding proteins, or initiation factors, potentially impacting translational regulation [111]. If inflammatory signalling is dysfunctional or prolonged, it may impair the translational machinery, increasing the likelihood of errors. The biochemical and physical properties of follicular fluid additionally affect translational control. Alterations in ionic composition, pH, and osmolarity can influence protein folding and the stability of ribosomes. Although these modifications are minor, they have the potential to significantly influence the efficiency and precision of protein synthesis over time. These microenvironmental elements may increase the likelihood of translational dysfunction in individuals as they age, a period during which compensatory mechanisms are limited [112].

The follicular microenvironment serves as a crucial upstream regulator of the oocyte’s translational integrity when considered comprehensively. The follicle modifies the molecular circumstances necessary for translation by adjusting redox balance, providing metabolic support, enhancing food availability, and modulating signalling pathways [113]. The decline in translational fidelity associated with ageing arises from disruptions in the environment that particularly affect particular components of the translational machinery, rather than merely compromising oocyte health overall [49]. This perspective highlights the influence of interactions with the surrounding somatic niche on the dynamic regulation of the oocyte’s translational decrease, which is not merely an inherent phenomenon. Understanding the follicular milieu as a regulator of translational integrity establishes a foundation for integrating systemic ageing, ovarian physiology, and the molecular mechanisms that govern oocyte competence. Furthermore, it asserts that instead of altering genetic material, therapies aimed at enhancing follicular metabolic and redox environments may elevate oocyte quality by preserving translational robustness. This technique aligns with the novel concept that oocyte ageing results from a confluence of external environmental stressors and internal molecular vulnerabilities [114].

## 9. Discussion

In our narrative review, we assert that the age-related deterioration of oocyte competence is mostly a gradual loss in translational fidelity, rather than solely attributable to chromosomal instability or mitochondrial dysfunction. Our comprehensive analysis reveals that oocyte ageing is marked by the selective impairment of maternal mRNA translation, inadequate temporal coordination of translation initiation and elongation, degradation of ribosome quality, and reduced integrity of the maternal proteome conveyed to the early embryo. We contend that translational dysregulation serves as a vital molecular axis connecting oocyte ageing to early embryonic arrest, even in chromosomally euploid embryos, by integrating findings from research on RNA-binding proteins, translation initiation factors, ribosome biogenesis, maternal mRNA clearance, redox regulation, and follicular microenvironmental signalling.

Takahashi et al. directly revealed that extensive disruption of ribosome loading on maternal transcripts correlates with maternal ageing, indicating that translational abnormalities emerge early and predate explicit meiotic or embryonic failure [74]. Duncan et al. independently documented age-related disruption of protein metabolism in oocytes, pinpointing ribosome-associated activities and proteostasis pathways as hitherto neglected aspects of reproductive ageing [25]. Potabattula et al. expanded these findings by illustrating age-related augmentations in ribosomal DNA methylation in human and mouse oocytes, suggesting epigenetic ageing of ribosome biogenesis pathways [26]. Takahashi et al. associated these worldwide alterations with reduced CPEB1 levels, whereas Sousa Martins et al. and Chen et al. demonstrated that CPEB1 operates within collaborative RNA-binding protein networks rather than as an independent regulator [14,19,74]. Collectively, these studies indicate that ageing affects both the molecular structure that maintains translational fidelity and capacity, as well as the translation process itself.

An essential finding from these investigations is the distinction between ribosome number and ribosome quality. Duncan et al. observed an increase in ribosome quantity and alterations in nucleolar architecture in old oocytes, while Potabattula et al. illustrated epigenetic remodelling of rDNA with age, indicating that ribosome synthesis may be quantitatively maintained or even augmented [25,26]. Takahashi et al. shown that ribosome loading becomes dysregulated, suggesting that an increase in ribosomes does not necessarily result in precise or prompt protein synthesis [74]. Iyyappan et al. underlined that throughout meiosis and early embryogenesis, translational control is both quantitative and qualitative, exhibiting oscillating patterns [16]. Collectively, these findings substantiate the concept that ageing facilitates the accumulation of ribosomes that are either physically or functionally impaired, hence augmenting translational noise instead of uniform translational failure.

Chen et al. illustrated the extremely selective and cell-cycle-synchronized recruitment of maternal mRNAs to polysomes during oocyte maturation, emphasising the essential function of RNA-binding proteins in temporal translation regulation [20]. According to Yang et al., DAZL serves as both a translational repressor and an activator, contingent upon transcript identity and developmental context, thereby introducing bidirectionality to translational regulation [13]. Sousa Martins et al. enhanced this model by illustrating that DAZL and CPEB1 collaboratively regulate maternal mRNA translation, with quantitative characteristics such as DAZL loading density and CPE structure determining translational efficacy [19]. Takahashi et al. contextualised this cooperative network within the framework of ageing by illustrating that diminished CPEB1 translation disrupts the repression activation equilibrium, leading to premature Cyclin B1 translation and postponed CDK1 activation [74]. Liu et al. included kinase signalling into this paradigm by elucidating a PGC7-AKT1-YBX1 axis that facilitates translational activation via the phosphorylation-dependent dissociation of YBX1 from eIF4E [22]. Collectively, these data indicate that ageing diminishes translational fidelity by disrupting RBP cooperation, post-translational modification, and developmental timing, rather than eradicating specific regulators.

Guo et al. established that selective translation during the oocyte-to-embryo transition necessitates the specialised cap-binding protein eIF4E1B, which specifically targets a subset of maternal transcripts enriched in CG-rich 5′UTR motifs [47]. Li et al. verified this finding, demonstrating that classical eIF4E activity is essential for a successful maternal-to-embryonic transfer and that the suppression of maternally stored eIF4E halts embryos at the two-cell stage [114]. Iyyappan et al. established initiation control within a dynamic framework by illustrating rhythmic activation of translation initiation and elongation throughout meiotic and early embryonic M-phases through the mTOR-4F-eEF2 axis [16]. Ming et al. expanded these findings by uncovering the postponed incorporation of stored maternal mRNAs into polysomes and identifying Eif1ad3 as a crucial initiation factor specific to the two-cell stage, vital for ribosome biogenesis and embryonic viability [23]. The data collectively indicate that translational start during early development is regulated by the deployment of stage-specific factors, rendering this phase particularly susceptible to age-related disturbances that affect temporal precision rather than overall translational capability.

Sha et al. revealed that during the human maternal-to-zygotic transition, specific maternal and zygotic decay pathways are utilised for the clearance of maternal mRNA, and that disruptions in these mechanisms are closely associated with developmental arrest in IVF embryos [18]. Chen et al. and Takahashi et al. offer indirect corroboration for this concept by demonstrating how erroneous translational suppression or premature activation modifies transcript destiny and protein longevity [20,74]. Ming et al. and Iyyappan et al. propose that translational engagement and transcript clearance are temporally coordinated processes rather than separate events [16,23]. These findings suggest that translational dysregulation linked to ageing may disrupt the interplay between translation and mRNA degradation, hence impacting protein synthesis and the seamless transfer of control from mother to embryo.

A further underexplored consequence of translational dysregulation is the emergence of low-frequency translational mistakes that disproportionately impact protein complexes necessitating precise stoichiometric equilibrium. Chen et al. and Yang et al. established that the disruption of translational time influences spindle-related genes, whereas Takahashi et al. associated the early translation of Cyclin B1 with modified cell cycle dynamics [20,60,74]. Age-related disturbances in proteostasis may lead to the accumulation of misfolded or dysfunctional proteins in ageing oocytes, as indicated by Duncan et al. Sha et al. further elucidate this by showing that early embryos possess a restricted ability to rectify molecular flaws inherited from the oocyte [18,25]. These findings endorse a concept wherein translational mistakes, despite their low frequency, can exert dominant-negative effects on spindle assembly, cytoskeletal architecture, and early embryonic development. This model provides a mechanistic elucidation for developmental failure in chromosomally euploid embryos that evade PGT-A detection.

Redox regulation is a significant modulator of translational integrity rather than a universal source of cellular harm. Petrova et al. demonstrated that developmentally regulated reactive cysteine alterations govern proteins critical for meiotic progression and early embryogenesis, establishing translation and proteome function as redox-sensitive processes [24]. Liu et al. demonstrated that YBX1-mediated translational repression is alleviated by phosphorylation-dependent processes potentially influenced by redox balance [111]. Convergent evidence from Duncan et al. and Potabattula et al. indicates that ageing oocytes subjected to metabolic and oxidative stress disrupt nucleolar function and ribosome synthesis [25,26]. Collectively, these findings suggest that reproductive ageing represents a transition from a tightly regulated physiological redox state to one characterised by instability in RBP function and translational machinery due to redox fluctuations.

Chen et al. identified the follicular niche as an upstream determinant of translational competence by demonstrating that signals coming from somatic cells regulate the translation of certain maternal mRNAs via paracrine activation of the PI3K-AKT-mTOR pathway [14]. Potabattula et al. demonstrated considerable inter-oocyte diversity in rDNA methylation within a single individual, indicating uneven epigenetic ageing among follicles [26]. Duncan et al. provide additional evidence for this notion by emphasising differences in oocyte translational activity and nucleolar structure [25]. Collectively, these findings elucidate the mechanistic basis for the clinical observation that oocytes retrieved from the same woman display heterogeneous developmental potential, attributable to localised variations in follicular microenvironmental support rather than consistent intrinsic ageing.

Ultimately, studies comparing people and mice indicate that human translational vulnerability is both heightened and preserved. Takahashi et al., Yang et al., and Chen et al. delineate conserved RBP and initiation driven translational pathways in murine oocytes, whereas Sha et al. and Potabattula et al. illustrate that analogous mechanisms function in human oocytes and embryos [18,20,26,60,74]. According to Potabattula et al., the translational and epigenetic ageing of ribosome biogenesis may be intensified by the prolonged meiotic arrest in humans, a phenomenon not entirely represented in short-lived model organisms [26]. This distinction suggests that translational integrity may be much more crucial to the success of human reproduction.

The molecular framework outlined in this review has direct implications for the interpretation of clinical outcomes in assisted reproduction and challenges prevailing paradigms that prioritize chromosomal integrity as the dominant determinant of embryo competence. The evidence synthesized here indicates that translational fidelity represents an independent and functionally critical layer of oocyte and embryo quality, which is not captured by conventional genetic or morphological assessments. As a result, current clinical strategies may systematically underestimate the contribution of non-genetic molecular defects to reproductive failure. Takahashi et al. demonstrated that age-associated translational dysregulation emerges before overt meiotic abnormalities, suggesting that molecular defects may already be embedded in oocytes that appear morphologically normal at retrieval [12]. Duncan et al. further showed that age-related alterations in protein metabolism and ribosome-associated pathways occur at the level of the growing oocyte, well before fertilization or embryo culture [25]. Potabattula et al. highlighted substantial inter-oocyte variability in ribosomal DNA methylation within the same individual, indicating that oocytes retrieved in a single stimulation cycle may differ markedly in translational competence despite comparable baseline clinical characteristics [26]. Together, these findings provide a molecular explanation for the frequent observation that only a subset of oocytes from a given cycle contributes to viable embryos.

One of the most immediate translational implications concerns the interpretation of euploid embryo failure following PGT-A. Chen et al., Yang et al., and Sousa Martins et al. demonstrated that disruption of translational timing affects spindle-associated and cytoskeletal transcripts without necessarily inducing chromosomal missegregation [19,20,60]. Takahashi et al. showed that premature Cyclin B1 translation alters meiotic timing rather than chromosome number [12]. Sha et al. demonstrated that embryos with intact chromosomal content may arrest due to defective maternal mRNA clearance [18]. Collectively, these studies indicate that euploid embryos may fail because of inherited defects in the maternal proteome, rather than because of undetected genetic abnormalities. This challenges the assumption that chromosomal normality equates to developmental competence and underscores the need for molecular biomarkers beyond ploidy status.

Translation-centered mechanisms also offer a framework for interpreting embryo kinetic data obtained through time-lapse imaging. Iyyappan et al. and Ming et al. demonstrated that translation during early development follows oscillatory and stage-specific patterns rather than continuous activity [16,23]. These findings suggest that deviations in cleavage timing may reflect underlying defects in translational coordination rather than nonspecific “poor embryo quality.” When viewed through this lens, abnormal morphokinetics may represent phenotypic readouts of impaired translation initiation, elongation, or protein availability. This interpretation supports the integration of kinetic data with molecular models of translation rather than relying on descriptive timing thresholds alone.

The follicular microenvironment emerges as a modifiable upstream determinant of translational integrity, with potential implications for ovarian stimulation strategies. Chen et al. demonstrated that somatic cell-derived signals regulate translation of specific maternal mRNAs via PI3K-AKT-mTOR signaling [20]. Petrova et al. showed that redox balance must be tightly regulated to support meiotic progression and early embryogenesis [24]. Duncan et al. and Potabattula et al. provided evidence that nucleolar function and ribosome biogenesis are sensitive to metabolic and oxidative conditions [26,77]. Together, these findings suggest that stimulation protocols, metabolic status, and follicular redox balance may influence oocyte quality at the level of translation, raising the possibility that targeted metabolic or redox modulation could preserve translational fidelity in selected patient populations.

From a translational research perspective, these insights open avenues for the development of novel biomarkers of oocyte and embryo competence. Sha et al. demonstrated that defects in maternal mRNA decay pathways correlate strongly with developmental arrest in human IVF embryos [18]. Potabattula et al. showed that rDNA methylation varies between oocytes and increases with age, providing a potential epigenetic marker of translational aging [26]. Liu et al. identified phosphorylation-dependent regulation of YBX1 as a critical switch for translational activation [115]. Collectively, these molecular features suggest that assessment of translational readiness, rather than static transcript abundance, may offer clinically relevant information about developmental potential. Importantly, the translational framework proposed here also carries implications for patient counseling and expectation management. The heterogeneity of translational aging within individual ovaries implies that reproductive potential cannot be inferred solely from chronological age or ovarian reserve markers. Duncan et al. and Potabattula et al. provide molecular support for the clinical observation that patients of similar age and reserve may exhibit markedly different outcomes [25,26]. Recognizing translational fidelity as a stochastic and follicle-specific property may help explain variable responses to treatment and reduce reliance on oversimplified prognostic indicators.

Finally, these findings underscore the need for a conceptual shift in how reproductive aging is addressed therapeutically. Rather than focusing exclusively on chromosomal screening or embryo selection, future strategies may need to consider interventions that preserve or restore translational robustness at the oocyte level. Such approaches could include optimization of follicular metabolic support, refinement of stimulation protocols to minimize oxidative stress, or targeted modulation of translational signaling pathways. While these strategies remain speculative, the molecular evidence reviewed here provides a strong rationale for expanding the clinical focus beyond genome integrity toward proteome quality and translational control. In summary, the translational landscape of the oocyte represents a critical and clinically relevant determinant of reproductive success. Integrating translational biology into assisted reproduction offers an opportunity to bridge the gap between molecular aging and clinical outcomes and may ultimately lead to more precise, mechanism-based approaches to infertility treatment. Finally, Table 3 summarizes how problems with translational regulation may explain common clinical observations and point the way for future translational strategies that will turn these molecular insights into useful ideas for assisted reproduction.

**Figure 1:**
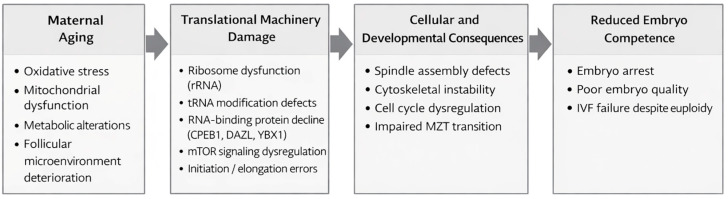
Mechanistic model of age-associated decline in translational fidelity and its consequences for oocyte competence and early embryo development. A mechanistic model illustrating the gradual decline in translational fidelity of the ageing oocyte. Maternal ageing and the consequent alterations in the follicular milieu induce molecular damage that accumulates and impacts ribosome integrity, transfer RNA modification, RNA-binding protein functionality, and translational signalling pathways. These disturbances diminish the precision of translation, hence altering the quantity and quality of the maternal proteome. Consequently, critical processes such as spindle formation, cytoskeletal organization, cell cycle progression, and the shift from maternal to zygotic development are all impacted.

Figure 1 shows a schematic summary of the proposed model. This gives a full visual picture of the molecular and cellular mechanisms talked about in this review.

## 10. Conclusions

Our research highlights the importance of translational integrity as a crucial, yet previously overlooked, element in evaluating oocyte and embryo competence. The data herein substantiates a theory suggesting that the gradual destabilisation of molecular systems regulating selective maternal mRNA translation, translation initiation timing, ribosome quality, and proteome integrity rather than solely chromosomal instability or mitochondrial dysfunction represents the principal mechanism of reproductive ageing. Even in the absence of explicit genetic anomalies, disruption of these mechanisms jeopardises the early embryo’s development by compromising the composition and functionality of the maternal proteome.

This review offers a cohesive mechanistic framework that connects oocyte ageing to early embryonic arrest by synthesising insights from RNA-binding protein networks, translation initiation and elongation pathways, ribosome biogenesis, maternal mRNA clearance, redox regulation, and follicular microenvironmental signalling. This idea clarifies the persistent failure of chromosomally euploid embryos in assisted reproduction, aligning genetic regularity with functional inadequacy.

Recognising translational fidelity as a crucial component of reproductive competence carries significant implications for both research and clinical practice. It emphasises the necessity of shifting from genome-focused evaluations to approaches that include translational robustness and proteome integrity. Thus, the development of molecular indicators and therapies that maintain or restore translational integrity in the oocyte and its supportive follicular environment may be crucial for future advancements in assisted reproduction.

## Figures and Tables

**Table 1:** Core evidence map of translational control across OET and MZT.

Study	System	Stage Focus	Main Molecular Axis	Primary Approach	Key Mechanistic Takeaway for This Review
Takahashi, et al. 2023 [12]	Mouse oocytes	Aging, meiotic re-entry	CPEB1, poly(A), ribosome loading	Ribosome loading, 3′UTR reporters, poly(A) assays, rescue/injection	Aging reduces CPEB1 and disrupts translation program, derepresses Ccnb1, mistimes CDK1 and meiosis
Guo, et al. 2023 [15]	Mouse oocytes/embryos	OET	eIF4E1B selective initiation	Maternal cKO, LACE-seq, proteomics	eIF4E1B selects CG-rich 5′UTR mRNAs; loss causes insufficient synthesis of competence proteins
Iyyappan, et al. 2023 [16]	Mouse oocytes/early embryos	Meiosis + first two mitoses	mTOR–4F–eEF2 oscillation	Genome-wide translatome profiling	Translational initiation/elongation oscillate with M-phase; timing reshapes polysome occupancy
Li, et al. 2021 [17]	Mouse embryos	MZT, early cleavage	eIF4E, 4E-BP1, mTOR	Genetic knockout, inhibitor 4EGI-1, mTOR inhibition	Maternal eIF4E supports early stages; eIF4E needed at 2-cell; mTOR regulates via 4E-BP1
Sha, et al. 2020 [18]	Human embryos	MZT, maternal mRNA clearance	M-decay, Z-decay, BTG4/CCR4-NOT, TUT4/7	Human preimplantation profiling	Defective maternal mRNA clearance correlates with developmental arrest in IVF embryos
Yang, et al. 2020 [13]	Mouse oocytes	Oocyte maturation	DAZL dual role	DAZL depletion, genome-wide ribosome loading, rescue	DAZL represses and activates distinct mRNA sets; loss disrupts loading and blocks maturation
Sousa Martins, et al. 2016 [19]	Mouse oocytes	Oocyte maturation	DAZL–CPEB1 synergy	Depletion, UTR mutagenesis, ribosome loading	DAZL cooperates with CPEB1; CPE architecture and DAZL loading determine activation strength
Chen, et al. 2013 [20]	Mouse oocytes with somatic cells	Maturation, competence	Somatic cues, PI3K–AKT–mTOR	Co-culture, AREG model, pathway modulation	Somatic EGF-like signals control translation of competence mRNAs via PI3K–AKT–mTOR
Chen, et al. 2011 [14]	Mouse oocytes/zygotes	Oocyte-to-zygote transition	DAZL, CPEB1 → DAZL synthesis	Polysome profiling, motif analysis	Translation is motif-driven; CPEB1 regulates DAZL; DAZL required for spindle formation and cleavage
Guo, et al. 2018 [21]	Mouse oocytes	Oogenesis, quality	MTOR stage-specific roles	Conditional knockouts	MTOR needed later for meiotic completion and embryonic competence; stage-specific effects
Liu, et al. 2024 [22]	Mouse oocytes	Translation activation	PGC7–AKT1–YBX1	Axis dissection, phosphorylation readouts	PGC7 scaffolds AKT1→YBX1 phosphorylation, releases YBX1 from eIF4E, activates Cyclin B1/YAP1 translation
Ming, et al. 2025 [23]	Mouse preimplantation	Preimplantation translation	Polysome dynamics, Eif1ad3	Fraction-resolved polysome profiling, multi-omics	Stage-specific delayed recruitment of stored mRNAs; Eif1ad3 translated at 2-cell, required for ribosome biogenesis
Petrova, et al. 2018 [24]	Drosophila + developmental redox	OET, early divisions	Redox cysteine switches	Proteome redox mapping	Developmentally controlled redox state modulates key proteins; ROS misregulation disrupts meiotic/embryo divisions
Duncan, et al. 2017 [25]	Mouse follicles/oocytes	Aging	Proteostasis, nucleolus, ribosome metabolism	Single-follicle RNA-seq, nucleolar markers	Aging alters nucleolar organization and protein metabolism pathways in highly translational oocytes
Potabattula, et al. 2022 [26]	Human + mouse GV oocytes	Aging	rDNA methylation	Single-oocyte bisulfite pyrosequencing	rDNA methylation increases with age; heterogeneity between oocytes suggests variable epigenetic aging

The included studies summarise the main idea that translational fidelity and selective translation control oocyte competence and early embryonic development. This table shows the main molecular axis, experimental setup, methodology, and main mechanistic contribution of each study.

**Table 2:** Age related perturbations affecting translational fidelity and proteome integrity.

Aging-Related Perturbation	Molecular Target	Mechanism	Predicted Consequence for Oocyte or Embryo
Reduced translational gatekeeping	CPEB1 network	Lower CPEB1 reduces repression and mistimes activation	Premature cell-cycle translation, altered meiotic timing
Proteostasis strain	Nucleolus, ribosome metabolism	Altered nucleolar markers and ribosome accumulation	Quantity may increase but fidelity and coordination degrade
Epigenetic aging of ribosome biogenesis	rDNA promoter/UCE methylation	Increased methylation with age; heterogeneity between oocytes	Variable translational competence within same cohort
Initiation and elongation desynchronization	mTOR-4F-eEF2 dynamics	Oscillatory regulation becomes vulnerable to stress	Stage-specific delays, reduced competence during OET/MZT
Impaired translation activation switches	AKT1-YBX1 interface	Failure of phosphorylation-dependent release of inhibition	Reduced translation of key targets during maturation
Redox imbalance	Redox-sensitive protein cysteines	Reactive cysteine changes alter protein function during OET	Defective meiosis and early embryonic divisions
Failure of maternal transcript clearance	M-decay and Z-decay	Abnormal persistence of maternal mRNAs in arrested embryos	ZGA disruption and developmental arrest

Mechanisms by which ageing and microenvironmental stress affect translational fidelity. This table links molecular disruptions to relevant data and expected developmental results.

**Table 3:** Clinical translational implications and actionable directions in ART.

Clinical Observation in ART	Translation-Centered Explanation	Practical Implication
Euploid embryos still arrest [18,20,60,74]	Non-genetic failure from defective maternal proteome, not captured by PGT-A	Add molecular competence layers beyond ploidy
Abnormal morphokinetics [16,23,114]	Stage-specific translation oscillation and delayed polysome recruitment	Interpret kinetics as proxy for translational coordination
Variable outcome within same cohort [25,26]	Heterogeneous epigenetic/translational aging among oocytes	Explains intra-cycle variability; supports individualized counseling
Microenvironment influences competence [24,47,116]	Somatic cues and redox window shape translational integrity	Optimize stimulation and follicular conditions, not only embryo selection
Potential biomarker paths [18,26,115]	Decay defects, rDNA methylation, translational activation switches	Targets for diagnostic research pipelines

Interpretation of ART clinical phenomena centered on translation and prospective biomarker or therapeutic methods developed from the incorporated mechanistic investigations.

## Data Availability

No new data were created or analyzed in this study. Data sharing is not applicable to this article.
